# 

**Published:** 1885-02

**Authors:** 


					﻿W HOU>Wt?M BER,
CCLXXIII.
FEBRUARY, 1885.
7.
X&Mite XXIV.
THE
BUFFALO
Medical and Surgical Journal.
E DITORS :
THO8. I.OTHROP, M. D.,
A. R. DAVIDSON, M.
Published by the Medical Journal Association.
No. 6 WEST CHIPPEWA ST.
Entered at the Post-Office at Buffalo, N. Y., as Second-class Mail Matter.
				

